# The cryo-electron microscopy supramolecular structure of the bacterial stressosome unveils its mechanism of activation

**DOI:** 10.1038/s41467-019-10782-0

**Published:** 2019-07-08

**Authors:** Allison H. Williams, Adam Redzej, Nathalie Rolhion, Tiago R. D. Costa, Aline Rifflet, Gabriel Waksman, Pascale Cossart

**Affiliations:** 1 Unité Biologie et Génétique de la Paroi Bactérienne, Institut Pasteur, Groupe Avenir, INSERM, 75015 Paris, France; 2 Institute of Structural and Molecular Biology, University College London and Birkbeck, London, WC1E 7HX UK; 3 Département de Biologie Cellulaire et Infection, Institut Pasteur, Unité des Interactions Bactéries-Cellules, 75015 Paris, France; 4 Inserm, U604, 75015 Paris, France; 5 INRA, Unité sous-contrat 2020, 75015 Paris, France; 6 Present Address: MRC Centre for Molecular Bacteriology and Infection, Department of Life Sciences, Imperial College London, London, UK

**Keywords:** Cryoelectron microscopy, Pathogens, Cryoelectron microscopy

## Abstract

How the stressosome, the epicenter of the stress response in bacteria, transmits stress signals from the environment has remained elusive. The stressosome consists of multiple copies of three proteins RsbR, RsbS and RsbT, a kinase that is important for its activation. Using cryo-electron microscopy, we determined the atomic organization of the *Listeria monocytogenes* stressosome at 3.38 Å resolution. RsbR and RsbS are organized in a 60-protomers truncated icosahedron. A key phosphorylation site on RsbR (T209) is partially hidden by an RsbR flexible loop, whose “open” or “closed” position could modulate stressosome activity. Interaction between three glutamic acids in the N-terminal domain of RsbR and the membrane-bound mini-protein Prli42 is essential for *Listeria* survival to stress. Together, our data provide the atomic model of the stressosome core and highlight a loop important for stressosome activation, paving the way towards elucidating the mechanism of signal transduction by the stressosome in bacteria.

## Abstract (web summary)

The bacterial stressosome is a large nanomachine and a key inducer of stress response. Here, the authors present the cryo-EM structure of the stressosome from the bacterial pathogen *Listeria monocytogenes* at 3.38 Å resolution and discuss its activation mechanism.

## Introduction

Microorganisms are constantly challenged by a variety of environmental stresses. In bacteria, the stressosome, a key inducer of the stress response is a very large nanomachine that responds to diverse environmental changes by triggering a protein partner-switching cascade of events, which culminate in the desequestration and activation of an alternative sigma factor. In Gram-positive organisms, sigma factor σ^B^^1,2^ activates at least 150 genes in response to a variety of stresses, which include, salt, ethanol, and blue light induced oxidative stress^1,3^. The stressosome was first identified in *Bacillus subtilis*. This cytoplasmic nanomachine, represents one of the largest bacterial molecular machineries identified^4^. However, its atomic structure and the mechanism of its activation have remain elusive.

The best studied stressosome model remains that of *Bacillus subtilis*. Its supramolecular structure was reported to consist primarily of RsbR, S, and T with a total molecular weight of 1.8 MDa^5,6^. A structural model of its assembly based on a low-resolution cryoelectron microscopy (cryo-EM) map was predicted almost a decade ago^5^. In this work, it was suggested that RsbR, RsbS, and RsbT assemble into a virus-like capsid with an atypical pseudo-icosahedral arrangement of the core structure, with sensory domains protrusions (or turrets)^5,7^. The amino acid sequence analysis of RsbR and RsbS reveals in both proteins the presence of a sulfate transporter and anti-sigma factor (STAS) domain, with RsbR containing at its N-terminus an additional domain termed “the non-heme globin” because of its structural similarity to the heme globulin fold superfamily proteins^1^. In the proposed model of the *B. subtilis* stressosome, the STAS domains of both proteins interact to form a core stressosome structure consisting of 10 dimers of RsbS, and 20 dimers of RsbR^5^. The atomic resolution structures of RsbR and RsbS are unknown, but that of the non-heme globulin domain of RsbR has been determined by X-ray crystallography^1^. In addition, STAS domain crystal structures of other non-stressosome proteins are available and have been used as starting models for homology modeling of RsbR and RsbS^1^. Interestingly, in *B. subtilis*, a number of paralogues of the RsbR sensor proteins that can substitute for RsbR have been identified^8^. Their function remains unclear, except for YtvA, which reportedly responds to blue light induced oxidative stress^1,3,9^. Although, uncharacterized, YtvA (Lmo0799) has also been identified in *Listeria monocytogenes*^10^. The structural key features identifying the mechanism of the stressosome activation and the partner-switching network are unknown. However, a number of biological studies primarily in *B. subtilis* have provided predictions as to how the stressosome might function. In the current model, the kinase RsbT is sequestered by the RsbR/RsbS stressosome core in the absence of stress. Upon an extracellular signal, an unknown mechanism triggers the sequential phosphorylation of RsbR (T171, T205) and RsbS (S59) by RsbT^1^. RsbT dissociation from the stressosome core then leads to the downstream σ^B^ cascade events^8,11–15^.

Recently, we showed that the turrets of the RsbR component of the *L. monocytogenes* stressosome interact with the N-terminus of Prli42, a membrane-bound mini-protein that is important for σ^B^ activation, bacterial growth, and for bacterial survival during oxidative stress^10^. Prli42 spans the membrane and interacts with the N-terminal domain of RsbR via its cytosolic N-terminal region^10^. It establishes a potential uncharacterized link between the stressosome and a membrane embedded receptor^10^.

Currently, there are no available atomic resolution structure of the stressosome from any organism. Here, in a first step toward understanding the molecular details of stressosome activation, we report an atomic resolution cryo-EM structure of the *L. monocytogenes* stressosome. Our data allowed the revelation of the atomic details of the complex arrangement between RsbR and RsbS in the larger context of the icosahedral assembly and further provides insights into the mechanism of stressosome activation and regulation. The structural analysis provided herein establishes the first step in the roadmap to understanding the structural mechanism underpinning the role of the stressosome in the bacterial stress response.

## Results

### Cryo-EM structure determination of the *Listeria* stressosome

**Fig. 1:**
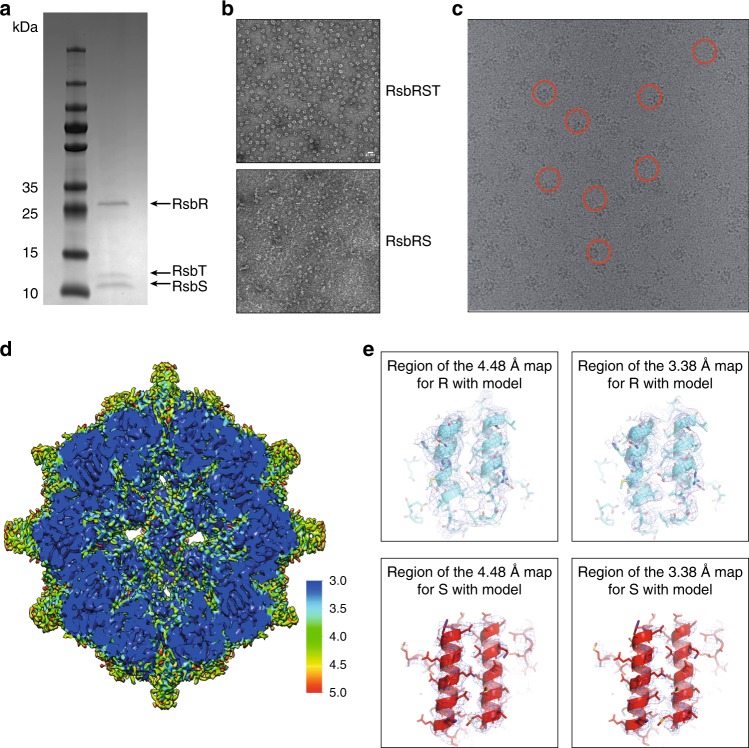
Cryo-EM structure determination of the *L. monocytogenes* structure. **a** SDS-PAGE analysis of the RsbR, S, and T complex. **b** Negative-stain electron microscopy analysis of the particles obtained by mixing either RsbR, S and T (left) or RsbR and S (right). **c** Cryo-EM micrograph of stressosome complexes. Some particles are shown in red circles. **d** Overall electron density of the 3.38 Å resolution map where densities outside of the core of the stressosome has been masked out. The map is contoured at a 3.0 sigma level with resolution mapped into it according to the side scale. **e** Overall quality of the electron density and fit of the model. Left and right panels: regions of the electron density for the 4.48 and 3.38 Å resolution maps shown in dark blue with final model shown in cartoon representation, respectively. Upper and lower panels: helices 169 Leu–219 Ser and 21 Asp–74 Ala of RsbR and RsbS, respectively

Fully assembled *L. monocytogenes* stressosomes were prepared from purified components as described previously^10^. RsbR and RsbS were first mixed in a molar ratio of 2:1 and then the preformed complex was combined with 10-fold excess of RsbT and 1 mM ADP to form the supramolecular complex. The stressosome was next purified by size-exclusion chromatography. SDS-PAGE (Fig. 1a) analysis confirmed our previously published results showing that the *L. monocytogenes* stressosome components RbsR, S, and T assemble in a stoichiometry of 2:1:1 similar to what has been reported for *B. subtilis*^6,10^. Here we demonstrate that all three proteins are needed in order to form the *L. monocytogenes* stressosome in vitro. Indeed *L. monocytogenes* RsbR and RsbS in the absence of RsbT did not form homogenous large assemblies when visualized by negative-stain electron microscopy, while in the presence of RsbT, homogenous particles are clearly visible (Fig. 1b).

The stressosome samples were vitrified on carbon coated holey grids and observed by cryoelectron microscopy (Fig. 1c). The data were collected on Titan Krios using the K2 direct electron detector, with an energy filter, in the counting mode. The data were processed using RELION 2.1^16^. Two maps of the *L. monocytogenes* stressosome complex were obtained. First, a 4.48 Å resolution map was determined with no symmetry applied (Table 1, Supplementary Fig. 1a). In this map the resolution in the core region of the stressosome extended to 3.5 Å, allowing the entire main chains of RsbR and RsbS to be traced and some of the side chains to be built. Next, to improve the resolution in the core structure, icosahedral symmetry (demonstrated by Marles-Wright et al.^5^ in their study of the *B. subtilis* stressosome core) was applied during the 3D refinement using a mask that excluded the turrets, yielding a 3.38 Å resolution map (Supplementary Fig. 1b, Table 1). In the latter map, the density for the C-terminal domain of RsbR is well defined, even for side chains, allowing the structure of this RsbR domain to be built. However, for RsbS, while the main chain was well defined, the density for the side chains was at times unclear due to averaging blurring out the side chains density in regions where the sequences of RsbS and RsbR diverge. These results can be rationalized by the fact that, in the stressosome, there are twice the number of RsbR dimers compared with RsbS dimers and that, along the threefold axis of the icosahedron, there are two and one RsbR and RsbS dimers, respectively. Therefore, we used the high-resolution map to trace the main chain and some side chains of RsbS, but we used the unaveraged map to complete the RsbS model. The known crystal structure of the N-terminal non-heme globin domain was docked into the turrets of the 4.48 Å map: the density in this region was, however, unclear and of not good enough quality to build and refine the existing model^7^. This might indicate that these domains are flexibly linked to the core. The final model contains all the residues of the RsbR and RsbS proteins, except for a short 13 amino acid flexible loop between residues 237 and 250 in the STAS domain of RsbR (see below). Noisy and patchy densities were observed for RsbT in the non-symmetrised map, and consequently, no model was built for that protein. Note that maps generated using D2 symmetry (suggested by Marles-Wright et al.^5^) do not show improvements in the density of the turrets or that of RsbT.

**Table 1:** Cryo-EM data collection, refinement, and validation statistics

	(EMDB-4508) (PDB 6QCM)	(EMDB-4509)
*Data collection and processing*		
Magnification	130,000	130,000
Voltage (kV)	300	300
Total electron dose (e^−^/Å^2^)	45	45
Defocus range (μm)	1.4 to −4.1 μm	1.4 to −4.1 μm
Pixel size (Å)	1.06	1.06
Symmetry imposed	C1	I1
Initial particle images (no.)	249,957	249,957
Final particle images (no.)	78,084	78,084
Map resolution (masked Å) Map resolution (unmasked Å) FSC threshold	4.48 6.32 0.143	3.38 3.60 0.143
*Refinement*		
Model resolution (Å) FSC threshold	4.22 0.5	
Model resolution range (Å)	3.0–5.8	
Map sharpening *B* factor (Å^2^) Model composition		
Non-hydrogen atoms Protein residues	52,618 7000	
*B* factors (Å^2^)		
Protein	62.10	
R.m.s. deviations		
Bond lengths (Å) Bond angles (°)	0.006 1.17	
Validation		
MolProbity score Clashscore Poor rotamers (%)	2.26 7.42 1.16	
Ramachandran plot		
Favored (%) Allowed (%) Disallowed (%)	84.49 15.17 0.34	

### The atomic structure of the *L. monocytogenes* stressosome

**Fig. 2:**
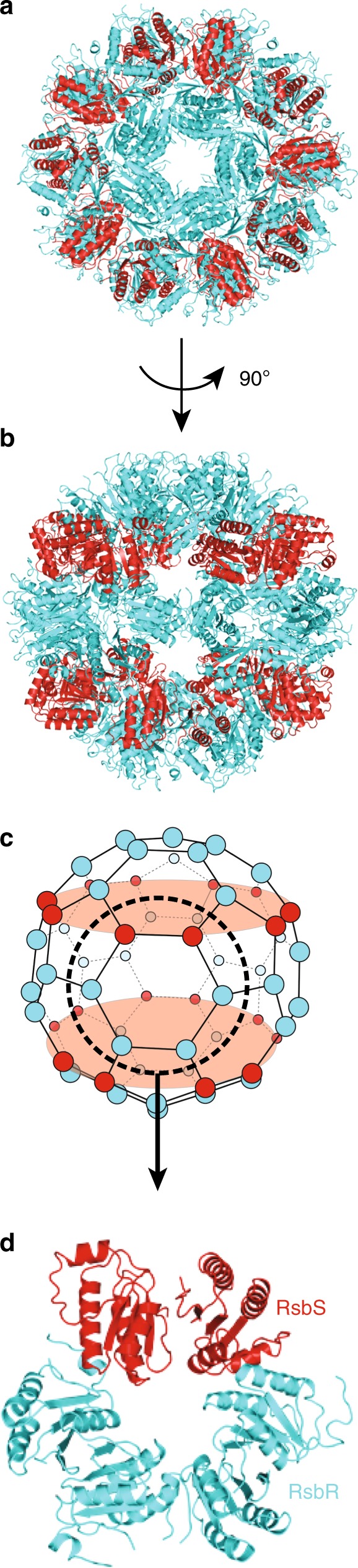
Overall structure of the core icosahedral structure of the stressosome. **a** View down the fivefold axis of the core icosahedral structure of the *L. monocytogenes* stressosome. Only the STAS domains of RsbR and RsbS are shown, in cyan blue and red ribbon representation, respectively. **b** Side view of the core icosahedral structure of the stressosome. This view is derived from the view in panel (**a**) by a 90° rotation along the horizontal axis. Representation of proteins is as in (**a**). **c** Schematic diagram of the core icosahedron (upper panel) and structure of the primary trimer of dimers (lower panel). In the upper panel, each protein is represented as a ball, cyan blue for RsbR and red for RsbS. The primary trimer of dimers of RsbR and RsbS onto which the entire structure is based is shown within the dashed circle, the molecular details of which are shown in the lower panel. **d** Structure of the primary trimer of dimers is shown in ribbon representation, with RsbR and RsbS color-coded cyan blue and red, respectively

The supramolecular arrangement of the *L. monocytogenes* stressosome is similar to that of *B. subtilis*: it is a truncated icosahedron comprised of 20 regular hexagons, 12 pentagons, 60 vertices, and 90 edges (Fig. 2). The stressosome contains 20 dimers of RsbR and 10 dimers of RsbS (Fig. 2a, b), arranged in repeating units of 2 dimers of RsbR and 1 dimer of RsbS. This hetero-hexagonal unit forms the basis on which the icosahedron is built. RsbS dimers are organized in two parallel planes within the icosahedron (shown in red in Fig. 2c), a configuration that might optimally support the structure. Here we report the experimentally derived atomic models of the stressosome components for which structures have not been reported, the C-terminal STAS domains of both RsbR and RsbS.

**Fig. 3:**
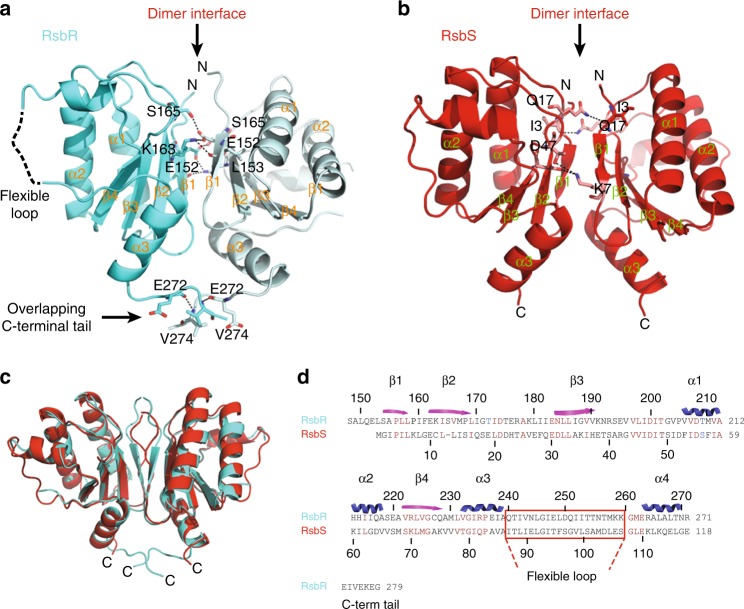
Structure of the STAS dimers of RsbR and RsbS. **a** Structure of the RsbR STAS dimer. The protein is shown in cyan blue ribbon representation. Residues participating in the dimer interface are shown in ball and stick representation with C, O, and N atoms color-coded in cyan blue, red, and dark blue, respectively. Residue numbers and secondary structure labels are provided, as well as the location of the dimer interface, the flexible loop and the C-terminal tail. **b** Structure of RsbS STAS dimer. The protein as well as interface residues are shown as in (**a**), except for the ribbon and the C atoms are color-coded red. **c** Superposition of the STAS domain structures of RsbR and RsbS. **d** Structure-based sequence alignment of RsbR and RsbS STAS domains. Residue numbers are provided for both proteins, as well as the location of the C-terminal tail of RsbR. Secondary structures are shown on top of the sequence alignment

The *L. monocytogenes* stressosome core proteins, RsbR and RsbS, display the signature β-strands-α-helical arrangement of a typical STAS domain where a central 4-stranded β-sheet is flanked by one α-helix on one side and three α-helices on the other (Fig. 3a, b). RsbR has a longer C-terminal sequence, absent in RsbS (Fig. 3c, d). This C-terminal tail between residues 272 and 279 plays a role in the formation of the interface between two RsbR subunits within the RsbR dimer (Fig. 3a). A superposition of RsbS and RsbR (Fig. 3c) yields a root mean square deviation in Cα atoms of 1.8 Å over 88 residues, demonstrating that the two proteins share very strong structural similarities in spite of a primary sequence similarity of only 25% (Fig. 3d).

The STAS domains of RsbR and RsbS each form head to head homodimers (Fig. 3a, b). The total buried surface area of the interface between the RsbR STAS domains within the RsbR dimer is 1027.3 Α^2^ while that within the RsbS dimer is 657.6 Α^2^. This difference in surface area buried upon dimerization is partly due to the fact that the C-terminal tails of the STAS domains in each RsbR subunit overlap to form a large interlocking interface, absent in RsbS (Fig. 3a, c). In both proteins, the bulk of the interactions within the dimer interface is formed by residues in two continuous β-strands labeled β1 and β2 in Fig. 3a, b. In addition, the interface of RsbR and RsbS dimers are held together primarily by hydrogen bonding and salt bridges formed between oppositely charged amino acid side chains (Fig. 3a, b).

**Fig. 4:**
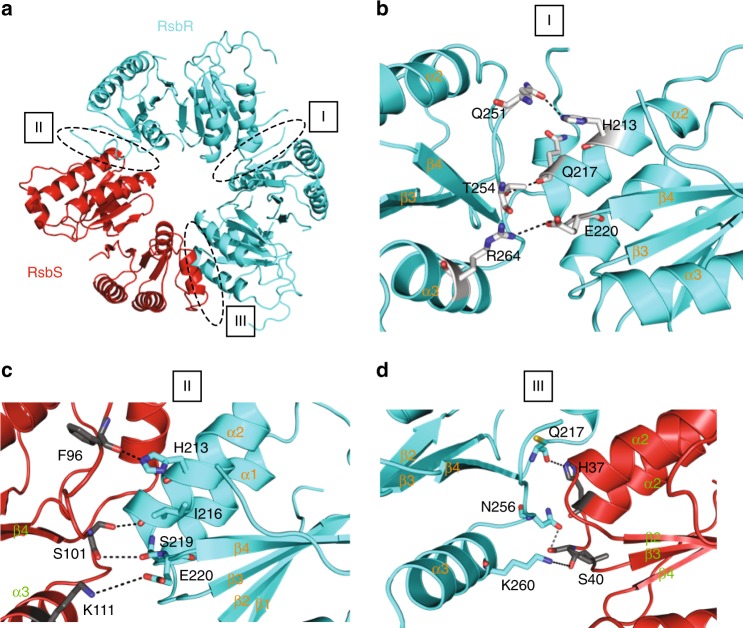
Details of the dimer–dimer interfaces. **a** Overall view of the primary trimer of dimers. Proteins are represented as in the lower panel of Fig. 2c. The labels locate the interfaces, the details of which are shown in panels (**b**–**d**). **b** Details of residues involved in dimer–dimer interface I. RsbR STAS domains are in ribbon representation color-coded cyan blue. Secondary structures involved in contributing residues to the interface are labeled. Residues involved in the interface are shown in ball and stick representation, with C, O, and N atoms color-coded in cyan blue, red, and dark blue, respectively. Residue identities and numbers are provided. **c** Details of residues involved in dimer–dimer interface II. Proteins are in ribbon representation color-coded cyan blue and red for RsbR and RsbS, respectively. Secondary structures involved in contributing residues to the interface are labeled. Residues involved in the interface are shown in ball and stick representation, with C, O, and N atoms color-coded in cyan blue for RsbR or red for RsbS, red, and dark blue, respectively. Residue identities and numbers are provided. **d** Details of residues involved in dimer–dimer interface III. Proteins, residues and labeling are as in panel (**c**)

Another aspect of the stressosome structure that was previously missing was the residue-specific structural characterization of the interactions between RsbR and RsbS subunits holding the stressosome together. Here we provide these details. The RsbR-RsbR and RsbR-RsbS interfaces maintaining the integrity of the icosahedral structure are between (i) two RsbR dimers and (ii) one RsbS dimer and two RsbR dimers (Fig. 4). All dimer–dimer interfaces, whether between RsbR dimers or between RsbR and RsbS dimers, involve residues in helical secondary structures and flexible loops (helix α2 and the flexible loop region between helices α3 and α4, for example, Fig. 4b–d). This is in contrast with the fact that interfaces within RsbR and RsbS homodimers are formed by residues in β-strands (Fig. 3a, b). These observations reveal that the interfaces between dimers might be less rigid than the ones within dimers, perhaps impacting on overall stressosome’s structural flexibility.

Overall, three interfaces bring the basic RsbR-RsbR-RsbS units together: one interface between two RsbR dimers (labeled “I” in Fig. 4a) and two interfaces between RsbR and RsbS dimers (labeled “II” and “III” in Fig. 4a). The buried surface area of the RsbR dimer–dimer interface is 578 Α^2^. Interestingly, interfaces I and II, have comparable interacting residues (Fig. 4b, c). In contrast, interface III is very different from interfaces I and II (Fig. 4d).

**Fig. 5:**
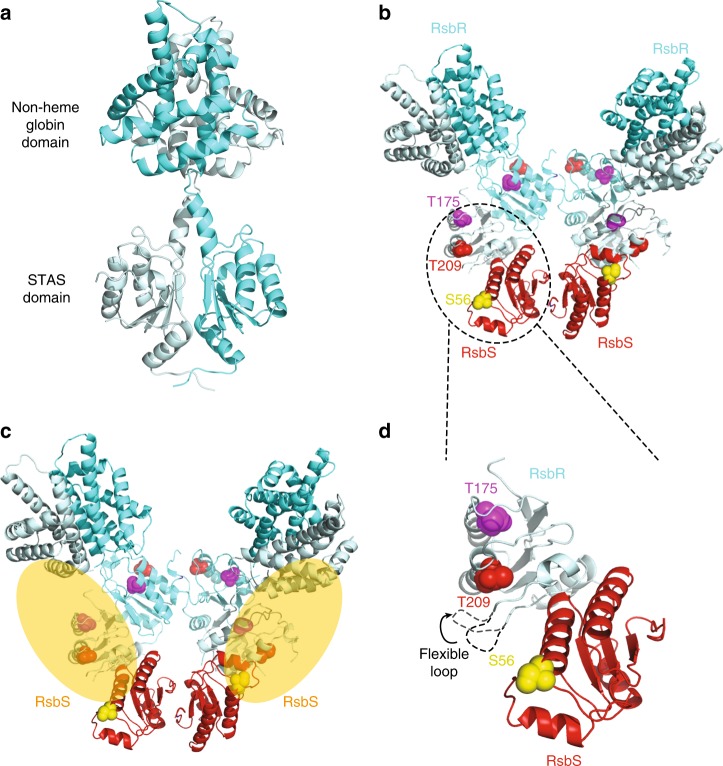
Mechanistic insights derived from the *L. monocytogenes* stressosome assembly. **a** Model of full-length RsbR dimer. Each subunit is shown in ribbon representation, color-coded in two hues of cyan blue (regular and pale). The two domains of RsbR are labeled. **b** Model of the primary trimer of dimers with the N-terminal domains of the RsbR subunits (the turrets) shown. Each subunit is shown in ribbon representation with the RsbR subunits shown as in panel (**a**) (cyan blue and pale cyan blue colors allow to distinguish between the two individual subunits within each dimer) and the RsbS subunits shown in red. Residues T175 and T209 of RsbR and S56 of RsbS are shown in sphere representation, color-coded magenta, red, and yellow, respectively. The dashed circle the region of the structure shown in panel (**d**). **c** Location of RsbT inferred from previous mutational studies and patchy densities in the *B. subtilis* and *L. monocytogenes* structures. RsbT is shown as a pale, semi-transparent, oval. The structure shown is the same as in panel (**b**). **d** Details of the interface between RsbR and RsbS emphasizing the proximity of T175, T209, and S56, as well as the disordered loop

While in the *L. monocytogenes* stressosome reconstruction presented here, the electron density for the STAS domain of RsbR and RsbS was of sufficient resolution and quality to build a complete model including side chains, the electron density for the turrets (Supplementary Fig. 1a) was of lesser resolution; however, the crystal structure of the *Bacillus* RsbR N-terminal domain was approximately docked into the globular, yet undefined, density of the turrets (Fig. 5a, b). Similarly, although some density that could be associated with RsbT was identified on the side of the RsbR N-terminal domain, it was sparse even in the *L. monocytogenes* higher-resolution maps, and no model for RsbT could be derived. In the predicted model of the *B. subtilis* stressosome, RsbT was also not visible but its position could be hypothesized based on previously published biochemical data^7^. Similarly, patchy density near the turrets was identified, a situation very similar to that observed here for the *L. monocytogenes* stressosome: thus, only the approximate position of RsbT could be inferred (Fig. 5c).

### Mutational analysis of RsbR and RsbS interacting region

In an effort to validate the structural assembly of the stressosome and to analyse the role of the interface of interaction between RsbR and RsbS and its role in stressosome activation, mutations were created and expressed from a non integrative plasmid: (a) one at the interface of RsbR-RsbS interaction (RsbR Q217L E220L T254A R264L), (b) one deleting the flexible loop region of RsbR (loop residues 237–251), and (c) one affecting the phosphorylation sites of RsbS and RsbR (RsbR T175A T209A, RsbS S56A). The Δ*rsbR* and Δ*rsbS* strains were viable and successfully transformed with plasmid expressing WT RsbR or WT RsbS. However, transformation with plasmid expressing either mutated *rsbR* or *rsbS* into Δ*rsbR* or Δ*rsbS* strains and even into WT *Listeria* was not possible. This indicates that these transformants are not viable and suggests a lethal phenotype or simply a toxic effect of the mutated proteins (Supplementary Table 1). These results suggest that in the absence of RsbR, the RsbR paralogues are able to substitute in the stressosome assembly. However, mutations at the interface of the interactions between RsbR and RsbS as well as deletion of the flexible loop in RsbR affect the survival of *Listeria*. Together, our data confirm that RsbR and RsbS are the major components of the stressosome core and that the flexible loop within RsbR, which partially occludes a key phosphorylation site (T209) plays a crucial role in the stressosome assembly.

### RsbR and Prli42 interaction is essential for *Listeria* survival

Previously we showed that Prli42, a membrane-bound mini-protein, tethers RsbR to the membrane^10^. We have hypothesized that Prli42 might be responsible for signal transmission and activation of the bacterial stress response. Based on our docking studies, one monomer of Prli42 is predicted to interact with one monomer of RsbR close to the dimer interface of N-terminal domain of RsbR (Supplementary Fig. 2). As previously described, the dimer interface of the N-terminal domain has an acidic patch that could be complementary (Supplementary Fig. 2) to the highly basic cytoplasmic N-terminal tail of Prli42 (Supplementary Fig. 2)^10^. In our previous study, independent mutations of each of the three highly basic residues (K4, K5, and R8) in the N-terminal tail of Pril42 affect binding to RsbR^10^. Based on our docking model (Supplementary Fig. 2), there are three glutamates (E109, E110, and E113) at the surface of the N-terminal domain of RsbR that could be complementary to the highly basic cytoplasmic N-terminal tail of Prli42 (Supplementary Fig. 2). To test the importance of these residues in the interactions with Prli42, E109, E110, and E113 were mutated to glutamines and pull down experiments were performed to test the binding of the RsbR variants to the Prli42. When these three residues E109, E110, and E113 of RsbR were mutated to glutamines (RsbRQQQ) a significant decrease in the amount of RsbR bound to Prli42 was observed illustrating the importance of these residues in the interaction between Prli42 and RsbR (Supplementary Fig. 3).

To determine the functional effects of the interaction between Prli42 and RsbR on bacterial viability following oxidative stress, *Listeria* or the mutants was exposed to hydrogen-peroxide, or, hydrogen-peroxide combined with iron to generate reactive oxygen species (ROS) via the Fenton reaction, and bacterial survival was determined by calculating relative colony-forming units (CFUs). In the presence of hydrogen-peroxide, the Δ*rsbR* and Δ*rsbS* were significantly more sensitive to oxidative stress when compared with WT (Supplementary Fig. 3b). The sensitivity of both mutants was restored to the WT levels after transformation with plasmid expressing WT RsbR or WT RsbS (Supplementary Fig. 3b). In the presence of combined hydrogen-peroxide and iron, the viability of Δ*rsbR* could be restored to wild WT via complementation with WT *rsbR*. However, RsbR-QQQ in the presence of combined hydrogen-peroxide and iron was not able to restore the viability of the Δ*rsbR* strain. Taken together our data suggest that Prli42 communicates with RsbR through the N-terminal domain of RsbR and that this interaction could be responsible for the communication of the stress signal to the protein.

## Discussion

The mechanism of stressosome activation in bacteria has not been elucidated. In a recent study, we identified a membrane protein, Prli42, capable of activating the stressosome of *L. monocytogenes* via direct interaction with the non-heme binding domain of RsbR, providing the first insights into the chain of events leading to stressosome activation by an extracellular stress signal^10^. We anticipated that the elucidation of the structure of the stressosome at atomic resolution would shed light on the mechanism of activation. Here we report such a structure for the *L. monocytogenes* stressosome and propose a mechanism for its activation.

The stressosome fundamental unit is a hetero-trimer of dimers with two RsbR dimers associating with one RsbS dimer. We show here not only that the structures of each individual RsbR and RsbS subunits are very similar, but also that the RsbR and RsbS dimers superimpose very well with each other. The major difference between the two is the presence of an additional C-terminal tail in RsbR which in the RsbR dimer forms an interlocking interface. This interface might play a role in the stability of the stressosome as a whole. While the interface between subunits in each dimer is primarily mediated by the rigid central β-sheet, the interface between dimers is supported by residues in α-helices and loops making these dimer–dimer interfaces more flexible. This flexibility is probably important for the partner-switching cascade that is essential for stressosome activation. Three interfaces between RsbR-RsbR and RsbS-RsbS dimers hold the icosahedron together: one between two RsbR-RsbR dimers and two between RsbR-RsbR and RsbS-RsbS dimers. Interestingly, there are shared features between the RsbR-RsbR dimer interface and one of the two interfaces between RsbR-RsbR and RsbS-RsbS dimers. However, the second interface between RsbR-RsbR and RsbS-RsbS dimers is different. This feature could be a safety net employed by *Listeria* that ensures that chimeras are not formed between monomers of RsbR and monomers of RsbS resulting in an aberrant stressosome assembly.

We have shown here that in the absence of RsbT the stressosome is not assembled. Clear and consistent density for RsbT was not observed and thus the structural role of RsbT in stressosome assembly could not be elucidated. However, a general shape and localization could be derived from previous biochemical studies and weak densities in the *B. subtilis* and the *L. monocytogenes* maps^5,6^. There are several potential interpretations for this observation. First, any structure outside the stressosome core might be flexible, or flexibly anchored to the core; this interpretation is consistent with our observation that the turrets are not as well defined in the electron density as the core is. Second, although 20 molecules of RsbT have been demonstrated to be part of the stressosome^6,10^ some of them might have dissociated during cryo-grid preparation; dissociation from macromolecular complexes upon cryo-freezing is a common problem in cryo-EM^17^. Third, RsbT might be partly disordered and may need to be activated before it attains its final folded conformation.

**Fig. 6:**
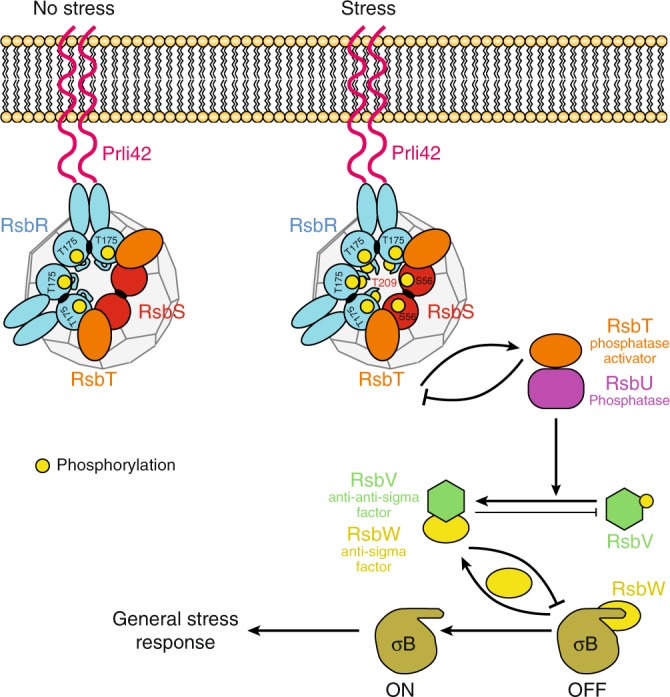
Proposed mechanism of stressosome activation. The stressosome is made up of RsbR, RsbS, and RsbT. Stress signals are transduced to the stressosome core from Pril42 to RsbR N-terminal regions (turrets) which could potentially undergo conformational changes. The conserved phosphorylation sites (T715, T209, S56) that activate the stressosome are clustered in one region. In cells that are unstressed, the serine-threonine kinase RsbT phosphorylates T175. During stress the phosphorylation of T175 enhances the activity of the RsbT kinase, which phosphorylates RsbS S56. Following phosphorylation of RsbS, RsbT is released, and binds and activate the phosphatase RsbU. RsbU dephosphorylates a form of RsbV, which releases RsbW. The anti-sigma factor RsbW binds to σB. Sigma B releases RsbW and activates RNA polymerase and the transcription of the stress response genes. During conditions of prolonged stress, RsbT phosphorylates residue T209 whose access is possibly controlled by a disordered loop. In *Bacillus* phosphorylation of residue T209 leads to mild activation of σB^12^

It is predicted that RsbT constitutively binds the stressosome and is only released in response to stress signal^18^. Concerning the mechanism of stressosome activation, there are conserved residues in RsbR (T175, T209) and RsbS (S56) (numbering here is after the *L. monocytogenes*) previously identified in *B. subtilis* as being important for phosphorylation by RsbT^1,4,5,8,12–14,19,20^. As shown in our structure, these conserved residues are located close to the surface of the stressosome, and thus are easily accessible for phosphorylation by RsbT (Fig. 5d). Under basal conditions, T175 in RsbR is constitutively phosphorylated. In our model, the phosphorylation sites of RsbR and RsbS are clustered at the dimer–dimer interface of RsbR and RsbS (Fig. 5d). Interestingly, T209 of RsbR a key residue in stressosome activation is located near the flexible loop between helices α3 and α4 (the α3–α4 loop) of RsbR between residue 237 and 251 region that is disordered (see above and Fig. 3a). This loop has the potential to prevent access of T209 to RsbT. Therefore, this loop could adopt two conformations, one (closed) preventing RsbT access to T209 and one (open) allowing access (Fig. 5d). The fact that it is disordered in our structure indicates that this loop may fluctuate between the two states. To test this hypothesis, we have deleted this loop and assessed the impact of this deletion on stressosome function. Remarkably the strain expressing RsbR without the loop was not viable. Thus, a plausible model for stressosome activation emerges (Fig. 6). Prli42 communicates with RsbR through its N-terminal domain, which may be responsible for the sensing or transmission of stress signals from the extracellular environment, thereby initiating the activation of the stressosome. In the structure presented herein, two of the three stressosome phosphorylatable sites, i.e., RsbR (T175) and RsbS (S56), are easily accessible by RsbT but the third one (RsbR T209) is not. Incidentally, RsbR T175 and RsbS S56 are responsible for stressosome activation under stress conditions. RsbR T209 resides near a loop (the α3–α4 loop, 237–250), and therefore, we hypothesize, that it might not be freely accessible by RsbT. Upon induction of a stress signal, RsbT binding to RsbR might be affected, leading to conformational changes that release the α3–α4 loop, thereby unmasking T209 that becomes accessible for phosphorylation by RsbT. Our structural data and model are supported by previous biological studies in *Bacillus* in which the equivalent of T209 (T205) is phosphorylated after extreme stresses and requires the absolute presence of RsbT^12^. It is also known that the phosphorylation of T209 limits the activation of the stress response. Therefore, it is plausible that T209 is partially protected from activation under normal conditions^1,12,14^. Thus, one additional effect of the flexibility of the α3–α4 loop is to position RsbT closer to residue S56 of RsbS, thereby making it a target for phosphorylation, which in turn leads to the destabilization of RsbT and its release. Current knowledge of the downstream events, suggests that RsbT would bind and activate the phosphatase RsbU^1,12,14^. RsbU dephosphorylates a form of RsbV, which in turn releases RsbW. The anti-sigma factor RsbW then binds to σB. Sigma B is released from RsbW and binds RNA polymerase and initiates the transcription of the stress response genes (Fig. 6).

In conclusion, we have biologically and structurally defined the stressosome nanomachinery and demonstrate the essentiality of the core structure for the survival of *Listeria*. We demonstrate that the N-terminal domain of RsbR is a stress sensing apparatus by virtue of its interaction with Prli42. Most importantly, our work has led to a plausible mechanism of activation potentially applicable to all Gram-positive bacteria stressosome apparatus. Taken together our study provides a snapshot of how stress signals are conveyed and then interpreted by the stressosome. Given that there are at least 20 dimers of RsbR, there are multiple potential ports of receiving diverse signals that can be fine-tuned in a single stress response leading to the activation of the stressosome. This machinery then, relays the signal(s) that initiates the phosphorylation-dependent partner-switching cascade, resulting in the activation of the alternative sigma factor σ^B^ that controls the transcription of more than 150 genes allowing the bacteria to adapt and response when exposed to external stress.

## Methods

### Protein purification

The stressosome proteins were purified as previously published with some modification^10^. Briefly, GST-RsbR, RsbS, and RsbT proteins were expressed in BL21 (DE3) Gold cells (Novagen), grown at 37 °C. All constructs were induced with 0.6 mM IPTG at an optical density at 600 nm (OD_600_) of 0.7 and collected after 12 h of induction at 16 °C. Cells were resuspended in 50 mM Tris pH 8.5, 150 mM NaCl, 1 mM DTT and lysed by sonication at 4 °C. Soluble fractions were recovered from cell debris by ultracentrifugation at 40,000 × *g*. After glutathione affinity chromatography and thrombin cleavage, proteins (RsbR, RsbS, and RsbT) were purified to homogeneity by size-exclusion chromatography using a Superdex 200 followed by a Superose 6 at 20 mM Tris pH 8.5, 1 mM DTT. Proteins were stored at 4 °C in preparation for protein complex assemblies.

### Stressosome assembly

The purified RsbR and RsbS were combined in a 1:2 ratio of RsbR to RsbS before being subjected to a gel filtration. The Superose 6 column was pre-equilibrated in 20 mM Tris pH 8.5, 1 mM DTT. Only fractions containing RsbR-RsbS proteins in the void volume fractions were pooled and concentrated by centrifugation using a 30 kDa molecular weight cutoff centrifugal filter (Vivaspin). Purified RsbR:RsbS complex was combined with an excess of RsbT and incubated at 4 °C for 4 h followed by gel filtration using a Superose 6 column (GE Healthcare) pre-equilibrated in freshly prepared 20 mM Tris HCl pH 8.5, 150 mM NaCl, 1 mM adenosine diphosphate, 1 mM DTT. Only those fractions containing RsbR-RsbS-RsbT proteins were collected.

### Electron microscopy

Three microliters of purified stressosome sample at 20 µg/ml concentration was applied to a glow discharged Quantifoil 2/2 +2 nm C grid, incubated for 1 min and plunged-frozen using a Vitrobot, with blot force set to 5, blotting time 4 s, humidity ~100% and at 4 °C. Grids were imaged in eBIC facility at the Diamond synchrotron using a Titan Krios operating at 300 kV. The data set was collected using K2 summit direct electron detector operating in counting mode with a Quantum energy filter at pixel size of 1.06 Å and a dose rate of ~2.25 electrons/Å^2^/s. For each 10 s exposure dose was fractioned into 20 movie frames. Defocus values in the data set ranged from −1.4 to −4.1 μm.

### Image processing

A total number of 3003 micrograph movies were aligned using MOTIONCORR 2.1^21^ and the CTF was estimated using CTFFIND4^22^. Approximately 100,000 particles were picked from the micrographs using GAUTOMATCH^23^ and an automatically generated Gaussian reference. All the following processing steps were done in RELION 2.1^16^ unless stated otherwise. After several rounds of 2D classification, the six best 2D classes were used to re-pick the particles from the micrographs. Approximately 250,000 particles were picked using GAUTOMATCH and these 2D classes as references. After several rounds of 2D classification ~88,000 particles from the best 2D classes were used to generate an initial model in cryoSPARC^24^ applying icosahedral symmetry. This model was low-pass filtered to 60 Å and used in 3D classification to further classify the particles. Approximately 78,000 particles belonging to the best 3D class were subjected to multivariate statistical analysis in Imagic-5^25,26^. Based on the Eigen vector analysis, we confirmed the conclusions of Marles-Wright et al.^5^ that the stressosome core has icosahedral symmetry. Next these particles were subjected to the 3D refinement. Applying icosahedral symmetry at that stage a map at 3.6 Å resolution was generated with a very well defined core. Post-processing in RELION using a mask that did not include the turrets resulted in a map at 3.38 Å resolution. Local resolution of the maps was calculated using RELION. In addition, a map of the stressosome was calculated at 6.32 Å resolution when no symmetry was imposed. Masking out the noise and sharpening of the map resulted with the final non-symmetrised map at 4.48 Å resolution.

### Model building and refinement

Building of stressosome STAS domains was carried out using Coot^27^, and restrained refinement was carried out using a combination of PHENIX^28^ and the CCP4 software suite^29^. MolProbity was used for structure improvements during building and refinement^30^. The available structure of the *B. subtilis* RsbR non-heme globin N-terminal domain (PDB entry code: 2BNL) was used to generate a homology model of the corresponding *L. monocytogenes* domain using Phyre 2^31^ and docked within the turret density using Rosetta 13. Accuracy of fit was evaluated using CHIMERA^32^. Initial docking model of Prli42 and RsbR was accomplished as previously described^10^. Initial docking of model of RsbR and Prli42 was created using AutoDock VinoDock^33^, GRAMM-X^34^, and FlexPepDock^35,36^ to predict potential binding modes. The final model of RsbR-Prli42 was positioned in the cryo-EM reconstruction of the RsbR in the stressosome at 3.38 Å. Iterative adjustment of the final docking model was made in COOT to ensure chemical accuracy.

### Bacterial strains and growth conditions

*Listeria monocytogenes* was grown overnight in Brain Heart Infusion (BHI) medium (Difco) at 37 °C while shaking at 200 rpm. When required, 7 µg/ml chloramphenicol or 5 µg/ml erythromycin were added to the culture medium. To generate *Listeria* mutants, standard techniques for DNA manipulation were used. RsbR or RsbS deletion mutants were constructed using the pMAD shuttle plasmid^37^ as described previously^38^ and confirmed by DNA sequencing. Integration of Prli42-Flag in the chromosome was verified by PCR using primers NC16 and PL95^39^. To obtain pP1-HA-RsbS WT, HA-tagged *rsbS* was amplified by PCR and cloned into the SalI/SmaI sites of pP1 (Supplementary Table 2). To obtain pP1-HA-RsbR WT and all the different point mutants (RsbR E109Q-E110Q-E113Q, RsbR T175A T209A, RsbR Δ237-251, RsbR Q217L E220L T254A R264L, and RsbS S56A), DNAs of the HA-tagged genes were synthetized (Gblock from Integrated DNA Technologies), then amplified by PCR and cloned into the SalI/SmaI sites of pP1 (Supplementary Table 2). Plasmids obtained in *E. coli* Top10 (One Shot™ TOP10 Chemically Competent *E. coli*, ThermoFisher) were verified by sequencing and were transformed into *L. monocytogenes* (Δ*rsbR*, Δ*rsbS* or WT) by electroporation. Strains, plasmids, and primers used in this study are listed in Supplementary Tables 2 and 3.

### Prli42-flag pull down and western blot

Interaction between HA-RsbR (WT or E118Q-E119Q-E122Q) and Prli42-flag was assessed by pull down experiments as previously described^10^. Briefly, pellets of 15 ml exponential cultures of *L. monocytogenes* EGD-e Δ*rbrR* HA-RsbR WT or E118Q-E119Q-E122Q, all co-expressing Prli42-flag, were washed with PBS. Cell pellets from 5 ml of each culture were lysed in 150 µl Tricine sample buffer (Biorad) supplemented with 20 mM DTT and 30 µl (corresponding to 10% of the pull-down input) was used for SDS-PAGE and immunoblotting of the input fraction. Cells from 10 ml of each culture were lysed and cell wall was digested with mutanolysin and Triton X-100. After sonication, the supernatant of both samples was recovered and 35 µl of settled M2 anti-flag beads (Sigma, washed three times with 1 ml of lysis buffer) were added to each lysate. Binding to the resin was performed overnight on a rotating wheel at 4 °C. Beads were collected by centrifugation, washed twice with lysis buffer and proteins were eluted in 70 µl Tricine sample buffer (Biorad) and incubation for 5 min at room temperature. Beads were removed, 20 mM of DTT were added and samples were run on 16.5% Mini-PROTEAN Tris-Tricine gels (Biorad). Protein detection was performed using standard protocols for membrane blocking and antibody incubation and proteins were revealed using Pierce ECL 2 Western Blotting Substrate (Fisher Scientific). Mouse monoclonal anti-flag (1/1000, M2, F3165, Sigma) and mouse monoclonal anti-HA antibodies (1/1000, 6E2, #2367, Cell Signaling Technology) were used as primary antibodies. Anti-mouse HRP-conjugated antibodies (1/10,000, AbCys) were used as secondary antibodies.

### H_2_O_2_ and FeC_6_H_5_O_7_ treatment

Survival to exposure to H_2_O_2_ and ferric citrate (FeC_6_H_5_O_7_) was assessed as described previously^10^. Overnight bacterial cultures were diluted 1/100 into 25 ml BHI and grown at 37 °C until exponential phase (OD_600_ of 1.0), after which 0.05% H_2_O_2_ (Sigma-Aldrich) or 2.5 µg/mL of FeC_6_H_5_O_7_ (Sigma), were added to all cultures. After 2 h, aliquots were collected, diluted and plated onto BHI plates to determine the number of colony-forming units (CFUs).

### Reporting summary

Further information on research design is available in the Nature Research Reporting Summary linked to this article.

## Supplementary information

Supplementary Information

Reporting Summary

Source Data

## Data Availability

The accession numbers for the density maps for the symmetric and asymmetric reconstructions reported in this paper are EMD 4508 and 4509. The accession number of the refined atomic model is 6QCM. The source data underlying Fig. 1a, and Supplementary Fig. 3a and b are provided as a Source Data file. Other data are available from the corresponding authors upon reasonable request.
