# Supplementary material for: The cryo-electron microscopy supramolecular structure of the bacterial stressosome unveils its mechanism of activation
Source: Nat Commun. 2019 Jul 8;10:3005. doi: 10.1038/s41467-019-10782-0 (PMC6614362; doi:10.1038/s41467-019-10782-0)
Supplement: Supplementary file 1 — Supplementary Information [file 41467_2019_10782_MOESM1_ESM.pdf]

Supplementary Information for:

The cryo electron microscopy supramolecular structure of the bacterial stressosome unveils its mechanism of activation

A. H. Williams, A. Redzej et al.

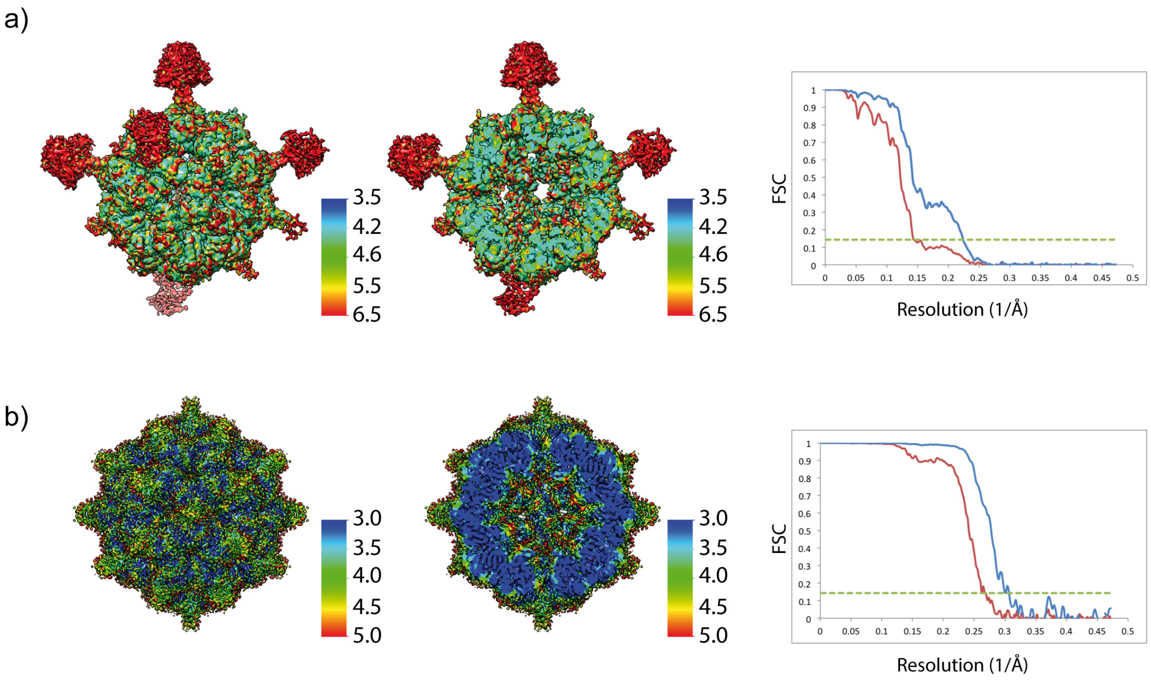

Supplementary Figure 1. Cryo-electron microscopy of the *L. monocytogenes* stressosome structure. a)Map of the *L. monocytogenes* stressosome complex achieved at 4.48 Å resolution when calculated with no symmetry imposed. b) Map of the *L. monocytogenes* stressosome complex achieved at 3.38 Å resolution when calculated with icosahedral symmetry imposed during the final reconstruction and a mask that excluded the turrets.

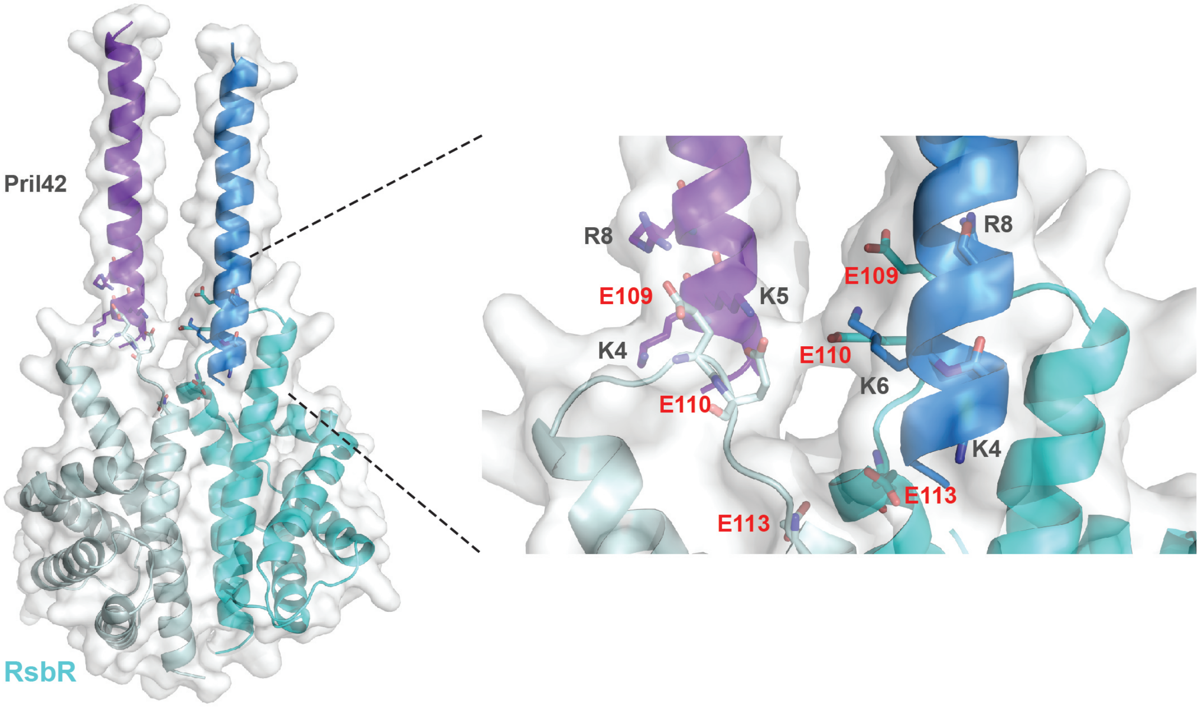

Supplementary Figure 2. Model of Prli42–RsbR interaction. Model displays one Prli42 per monomer of RsbR.

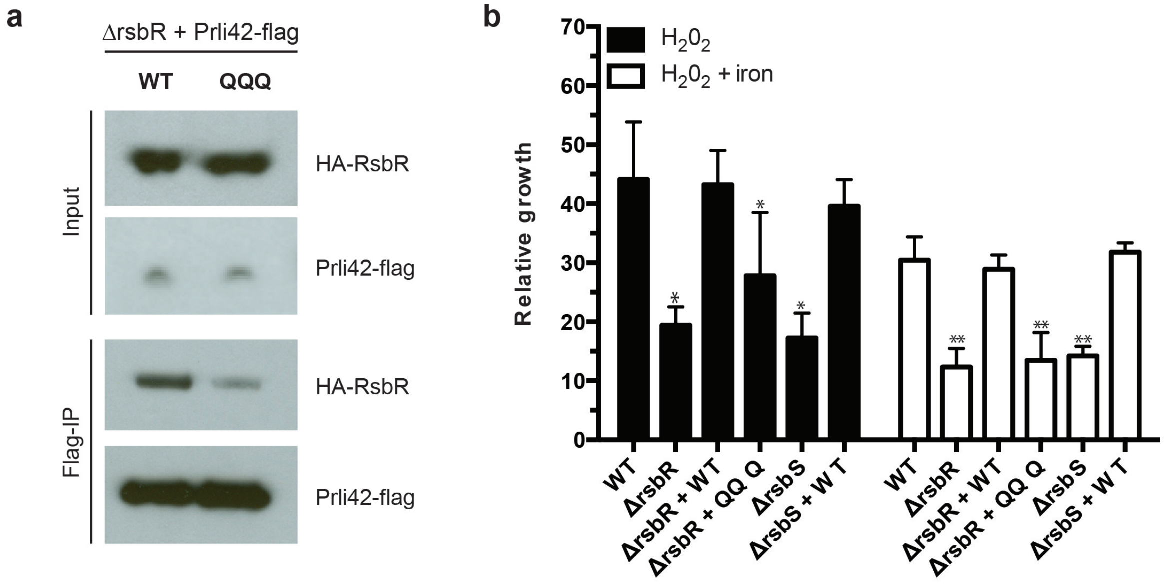

Supplementary Figure 3. Interaction of Prli42 with RsbR-QQQ and sensitivity of strains to ROS-generating conditions. **a**, Pull down of HA-RsbR and Prli42-flag. WT and E109Q-E110Q-E113Q variants of HA-RsbR were co-expressed in the RsbR deletion strain together with Prli42-flag, flag-pull down followed by immunoblotting against the HA-tag. **b**, Sensitivity of *L. monocytogenes* ΔrsbR complemented with variants of RbsR to oxidative stress. Bacteria were grown in BHI medium until exponential phase and 0.05% H_2O_2 +/- iron (2.5 ug/mL Ferric citrate) were added for 2h. The number of viable bacteria was monitored by CFUs. For each strain, the number of CFUs is shown relative to the number of CFUs at time zero. Results are expressed as mean ± SEM of three independent experiments.

	Strains			
**Constructs**	*E. coli*	EGD-e WT	EGD-e Δ*rsbR*	EGD-e Δ*rsbS*
no plasmid	+	+	+	+
pP1-HA RsbR WT	+	+	+	ND
pP1-HA RsbR T175A T209A	+	-	-	ND
pP1-HA RsbR Δ237-251	+	-	-	ND
pP1-HA RsbR Q217L E220L T254A R264L	+	-	-	ND
pP1-HA RsbR E109Q E110Q E113Q	+	ND	+	ND
pP1-HA RsbS WT	+	+	ND	+
pP1-HA RsbS S56A	+	-	ND	-

Supplementary Table 1: Summary of constructs and their ability to rescue or induce lethal phenotypes in different strains of *Listeria.*

Primer	Sequence
**Oligonucleotides used to create mutant**	
rsbR-Up-F	GGAAGATCTCTGATGGTGAAGCGTGGTGACG
rsbR-Up-R	CAGTTATTTTCCAACCTTTCTCCAC
rsbR-Down-F	GTGGAGAAAGGTTGGAAAATAACTGATACTGTGGGGATACCAATCTTAAAG
rsbR-Down-F	CGGCCATGGCCTGGAAGACCTGCTCCAAGACC
rsbS-Up-F	CGGCCATGGAGCAGACTTACTGAATGACTGG
rsbS-Up-R	AGTATTCACCCCTCTTTTTCTACTATCTC
rsbS-Down-F	GAGATAGTAGAAAAAGAGGGGTGAATACTACATGACATTCCAATCCTGTG
rsbS-Down-F	CGCGGATCCCCGTATAGTTCAAGAACCGAGC
**Oligonucleotides used to amplify synthetic DNA**	
pP1-HA-RsbR F	TCCCCCGGGATGTATCCATATGATGTTCC
pP1-HA-RsbR R	ACGTGTCGACTCACCCCTCTTTTTCTACTATCTC
**Oligonucleotides used to amplify RsbS from gDNA**	
pP1-HA-RsbS F	TCCCCCGGGATGTATCCATATGATGTTCCAGATTATGCTGGGATACCAATCTTAAAGTT AGGTG
pP1 HA-RsbS R	ACGTGTCGACTCATTCCCCCAATTCCTGTTTAAG
**Verification of pAD integration in the *Listeria* chromosome**	
NC16	GTCAAAACATACGCTCTTATC
PL95	ACATAATCAGTCCAAAGTAGATGC

Supplementary Table 2: Summary of primers utilized in this study

Strains or plasmids	Characteristics	Collection no.	Source or reference
**Strains**			
EGDe	*Listeria monocytogenes* WT strain	BUG1600	Mackanes et al 1964
EGDe D*rsbR*	*Listeria monocytogenes* EGDe *rsbR* deletion mutant	BUG4256	this study
EGDe D*rsbS*	*Listeria monocytogenes* EGDe *rsbS* deletion mutant	BUG4257	this study
EGDe D*rsbR* + HA-RsbR	EGDe D*rsbR* harboring pP1-HA-RsbR WT	BUG4258	this study
EGDe D*rsbR* + HA-RsbR E118Q-E119Q-E122Q	EGDe D*rsbR* harboring pP1-HA-RsbR E118Q-E119Q-E122Q	BUG4259	this study
EGDe D*rsbS* + HA-RsbS	EGDe D*rsbS* harboring pP1-HA-RsbS WT	BUG4260	this study
EGDe D*rsbR* Prli42-FLAG + HA-RsbR	pAD-Prli42 WT-FLAG chromosomally integrated in EGDe D*rsbR* harboring pP1-HA-RsbR WT	BUG4261	this study
EGDe D*rsbR* Prli42-FLAG + HA-RsbR E118Q-E119Q-E122Q	pAD-Prli42 WT-FLAG chromosomally integrated in EGDe D*rsbR* harboring pP1-HA-RsbR E118Q-E119Q-E122Q	BUG4262	this study
EGDe D*prli42*	*Listeria monocytogenes* EGDe *prli42*deletion mutant	BUG3588	Impens et al., 2017
**Plasmids**			
pMAD	shuttle vector used for cretaing plasmid for mutagenesis	BUG1957	Arnaud et al., 2004
pMAD-*rsbR*-UD	plasmid used to create the D*rsbR* mutant	BUG4240	this study
pMAD-*rsbS*-UD	plasmid used to create the D*rsbS* mutant	BUG4255	this study
pAD-Prli42-FLAG	integrative plasmid to create the Prli42-FLAG strains	BUG3948	Impens et al., 2017
pP1	replicative plasmid carrying a strong constitutive promoter cloned in the EcoRI site	BUG0942	Dramsi et al., 1995
pP1-HA RsbR	pP1 with N-terminal HA-tagged *rsbR* cloned into SalI and SmaI sites	BUG3999	this study
pP1-HA RsbR E118Q-E119Q-E122Q	pP1 with N-terminal HA-tagged *rsbR* E118Q-E119Q-E122Q cloned into SalI and SmaI sites	BUG4263	this study
pP1-HA RsbR T172A T209A	pP1 with N-terminal HA-tagged *rsbR* T172A T209A cloned into SalI and SmaI sites	BUG4264	this study
pP1-HA RsbR D237-251	pP1 with N-terminal HA-tagged *rsbR* D237-251 cloned into SalI and SmaI sites	BUG4265	this study
pP1-HA RsbR Q217L E220L T254A R264L	pP1 with N-terminal HA-tagged *rsbR* Q217L E220L T254A R264L cloned into SalI and SmaI sites	BUG4266	this study
pP1-HA RsbS	pP1 with N-terminal HA-tagged *rsbS* cloned into SalI and SmaI sites	BUG4267	this study
pP1-HA RsbS S56A	pP1 with N-terminal HA-tagged *rsbS* S56A cloned into SalI and SmaI sites	BUG4268	this study

Supplementary Table 3. Strains and plasmids used in this study
